# Hormone therapy and cancer risks in transgender people: a systematic review

**DOI:** 10.1590/S2237-96222024v33e2024319.especial.en

**Published:** 2025-01-10

**Authors:** Liza Yurie Teruya Uchimura, Tatiana Yonekura, Mabel Fernandes Figueiró, Jeane Roza Quintans, Patrícia Freire, Fernando Henrique de Albuquerque Maia

**Affiliations:** 1 Hospital do Coração, São Paulo, SP, Brazil; 2 Fundação de Ensino e Pesquisa em Ciências da Saúde, Brasília, DF, Brasil; 3 Ministério da Saúde, Secretaria de Atenção Especializada à Saúde, Brasília, DF, Brazil; 4 Universidade de São Paulo, Faculdade de Medicina, SP, Brazil

**Keywords:** Personas Transgénero, Neoplasias Malignas, Hormonal, Hormonoterapia, Revisión Sistemática, Transgender People, Malignant Neoplasms, Hormonal, Hormone Therapy, Systematic Review

## Abstract

**Objective:**

To identify the available evidence on the risk of developing cancer in transgender people undergoing hormone therapy.

**Methods:**

This was a rapid systematic review conducted in the PubMed, Embase, Virtual Health Library, Cochrane Library and Epistemonikos databases. Screening and data extraction were performed by independent reviewers using the Rayyan platform. Data extraction was carried out by 3 independent reviewers. We used the Joanna Briggs Institute checklists specific to cohort and case-control studies to assess the methodological quality of the included studies.

**Results:**

Five studies were included, 4 cohort studies and 1 case-control. The risk of transgender people developing cancer while on hormone therapy was identified by 2 studies and not identified in 3 studies.

**Conclusion:**

Despite studies with large sample sizes and rigorous selection criteria, the literature does not present a consensus on the association between hormone therapy and the development of cancer in transgender people.

## Study contributions

**Main results:**

Five studies published between 2013 and 2021 were identified. The risk of developing cancer in transgender people undergoing hormone therapy was found in two studies, but there is no clear consensus for recommendations.

**Implications for services:**

It contributes to comprehensive healthcare for transgender people. It supports more informed decision-making in the hormone therapy process and enhances the options available within the healthcare network for this population.

**Perspectives:**

The review highlights the need for more randomized, long-term studies to assess the risks and benefits of hormone therapy in transgender people, in different subgroups, such as age, duration of use and types of hormones.

## RESUMEN

**Objetivo:**

Identificar las evidencias disponibles sobre el riesgo de desarrollar cáncer en personas transgénero que reciben tratamiento con hormonoterapia.

**Métodos:**

Se trata de una revisión sistemática rápida en las bases de datos PubMed, Embase, Biblioteca Virtual en Salud, Cochrane Library y Epistemonikos. La selección y extracción de datos fueron realizadas por revisores independientes en la plataforma Rayyan. La extracción de datos fue llevada a cabo por tres revisores independientes. Utilizamos las listas de verificación del Instituto Joanna Briggs específicas para estudios de cohortes y casos y controles para evaluar la calidad metodológica de los estudios incluidos.

**Resultados:**

Se incluyeron cinco estudios, de los cuales cuatro fueron estudios de cohortes y uno fue un estudio de caso y control. El riesgo de desarrollar cáncer en personas transgénero bajo hormonoterapia fue identificado en dos estudios y no identificado en tres estudios.

**Conclusión:**

A pesar de la existencia de estudios con muestras grandes y criterios de selección rigurosos, la literatura no presenta un consenso sobre la asociación entre la hormonoterapia y el desarrollo de cáncer en individuos transgénero.

## INTRODUCTION

The concept of transgender or gender nonconforming refers to individuals whose gender identity differs from that assigned at birth.^1, 2^ This divergence may lead to gender transition, which includes hormone therapy and gender affirming surgeries, aimed to align the body with the individual’s gender identity. Approximately 0.6% of the United States population identifies as transgender.^3^ In Brazil, about 2% of the population is transgender.^4^

The World Professional Association for Transgender Health emphasizes the need for individualized clinical care for transsexual, transgender, and gender-nonconforming individuals. In line with the association’s guidelines, which stress the importance of personalized care, studies have shown that gender-affirming surgery yields better outcomes when performed by multidisciplinary teams specialized in transgender healthcare.^5^

The National Policy for Comprehensive Health of Lesbians, Gays, Bisexuals, Transvestites and Transsexuals^6^ and the guidelines for the transsexualization process in the Brazilian National Health System^7^ highlight the fundamental need to guarantee access to outpatient care for transgender individuals undergoing the transsexualization process. This underscores the importance of multidisciplinary work within the Brazilian National Health System.^6- 8^

The number of services and health professionals capable of providing comprehensive health care for transgender people remains insufficient, which demonstrates a gap in healthcare for this population in the country. As of 2022, only 11 services in Brazil were accredited to provide specialized care within the transsexualization process. In 2023, an additional 10 services were accredited.^9^

Hormone therapy enhances secondary sexual characteristics to align with an individual’s gender identity. Transgender individuals may receive steroid hormones, prescribed by healthcare professionals, to reduce psychological distress and induce desired physical changes such as reduced skin oiliness, changes in body fat distribution, and hair growth/loss.^10^ Gender-affirming hormone therapy may be administered in high doses over several decades^3^

The potential cancer risk associated with hormonal therapy in transgender individuals is a significant concern.^11^ This potential risk is often used as a justification for contraindicating or discontinuing hormone therapy. The use of hormones, especially estrogen, may influence the development of cancer cells, which increases the risk of certain types of cancer, such as breast cancer. It is important to note that the risk varies according to several factors, including the type of hormone, the dose and the duration of treatment. Other risk factors, such as family history and lifestyle habits, can also contribute to the development of the disease.

The benefits of hormone therapy for the mental health and well-being of transgender people are undeniable. Identifying cancer risks is crucial for guiding, shaping, and informing public health policies and raising awareness of the health issues affecting transgender individuals.

This rapid systematic review aims to identify the available evidence on the risk of developing cancer in transgender people undergoing hormonal therapy.

## METHODS

This was a rapid review, following the steps outlined by the Cochrane Rapid Reviews Methods Grou^p^ 12 to investigate the risk of developing cancer in transgender people undergoing hormone therapy. The rapid review methodology aims to enhance the efficiency of conducting the review while seeking to minimize the impact on the study’s quality and bias.^13^ The research question of this review was constructed based on the PECOS framework (population, exposure, comparator, outcomes and type of study) (Box 1). This rapid review was focused on the question: What is the risk of cancer development in transgender individuals using hormone therapies?

**Box 1:** Research question structured by population, exposure, comparator, outcomes and study types

P	Transgender people
E	Hormone therapy (progesterone, testosterone, estrogen)
C	People without hormone therapy
O	Risk of developing any type of cancer
S	Systematic review, cohort study, non-randomized clinical trials, randomized clinical trials and case-control studies

Methodological shortcuts included limiting the review to studies published in Portuguese, English, or Spanish, and conducting a quality assessment by one reviewer, later verified by a second. The protocol was registered on the Zenodo.org repository before study selection began.

The literature search was performed in October 2023 across PubMed, Embase, Cochrane Library, the Virtual Health Library (VHL), and Epistemonikos, without date restrictions. The search strategies were based on controlled vocabularies, including Medical Subject Headings (MeSH) from PubMed, Emtree from Embase, and Health Sciences Descriptors (DeCS) from Virtual Health Library (VHL). Uncontrolled terms, such as keywords and synonyms, were also used in developing the strategies (Box 2).

**Box 2:** Search strategy and results obtained from each bibliographic database

Database	Search strategy	Results
**PubMed**	(((“Transgender Persons”[Mesh] OR Person, Transgender OR Transgender Person OR Transgenders OR Transgender OR Transgendered Persons OR Person, Transgendered OR Persons, Transgendered OR Transgendered Person OR Two-Spirit Persons OR Person, Two-Spirit OR Two Spirit Persons OR Two-Spirit Person OR Transsexual Persons OR Person, Transsexual OR Transsexual Person OR Transsexuals OR Transsexual) OR (“Transsexualism”[Mesh] OR Transgenderism)) AND ((((“Hormone Replacement Therapy”[Mesh] OR Therapy, Hormone Replacement OR Hormone Replacement Therapies OR Replacement Therapies, Hormone OR Therapies, Hormone Replacement OR Replacement Therapy, Hormone) OR (“Testosterone”[Mesh] OR “Testosterone therapy”)) OR (“Progesterone”[Mesh] OR “Progesterone therapy”)) OR (“Estrogen Replacement Therapy”[Mesh] OR Estrogen Replacement Therapies OR Replacement Therapies, Estrogen OR Therapies, Estrogen Replacement OR Therapy, Estrogen Replacement OR Replacement Therapy, Estrogen OR Estrogen Replacement OR Estrogen Replacements OR Replacements, Estrogen OR Replacement, Estrogen OR Estrogen Progestin Replacement Therapy OR Estrogen Progestin Combination Therapy)) ) AND (“Neoplasms”[Mesh] OR Tumor OR Neoplasm OR Tumors OR Neoplasia OR Neoplasms OR Cancer OR Cancers OR Malignant Neoplasm OR Malignancy OR Malignancies OR Malignant Neoplasms OR Neoplasm, Malignant OR Neoplasms, Malignant OR Benign Neoplasms OR Benign Neoplasm OR Neoplasms, Benign OR Neoplasm, Benign)	139
**Cochrane Library**	1 MeSH descriptor: [Transgender Persons] explodes all trees #2 (Transgender Person OR Transgenders OR Transgender OR Transgendered Persons OR Person, Transgendered OR Persons, Transgendered OR Transgendered Person OR Two-Spirit Persons OR Person, Two-Spirit OR Two-Spirit Persons OR Two-Spirit Person OR Transsexual Persons OR Person, Transsexual OR Transsexual Person OR Transsexuals OR Transsexual ): ti ,ab,kw #3 MeSH descriptor: [Transsexualism] explodes all trees #4 (Transsexualism OR Transgenderism ): ti ,ab,kw #5 MeSH descriptor: [Hormone Replacement Therapy] explodes all trees #6 (Therapy, Hormone Replacement OR Hormone Replacement Therapies OR Replacement Therapies, Hormone OR Therapies, Hormone Replacement OR Replacement Therapy, Hormone ): ti ,ab,kw #7 MeSH descriptor: [Testosterone] explodes all trees #8 (“Testosterone therapy” ): ti ,ab,kw #9 MeSH descriptor: [Progesterone] explodes all trees #10 (“Progesterone therapy” ): ti ,ab,kw #11 MeSH descriptor: [Estrogen Replacement Therapy] explodes all trees #12 (Estrogen Replacement Therapies OR Replacement Therapies, Estrogen OR Therapies, Estrogen Replacement OR Therapy, Estrogen Replacement OR Replacement Therapy, Estrogen OR Estrogen Replacement OR Estrogen Replacements OR Replacements, Estrogen OR Replacement, Estrogen OR Estrogen Progestin Replacement Therapy OR Estrogen Progestin Combination Therapy ): ti ,ab,kw #13 MeSH descriptor: [Neoplasms] explodes all trees #14 (Tumor OR Neoplasm OR Tumors OR Neoplasia OR Neoplasms OR Cancer OR Cancers OR Malignant Neoplasm OR Malignancy OR Malignancies OR Malignant Neoplasms OR Neoplasm, Malignant OR Neoplasms, Malignant OR Benign Neoplasms OR Benign Neoplasm OR Neoplasms, Benign OR Neoplasm, Benign ): ti ,ab,kw #15 #1 OR #2 OR #3 OR #4 #16 #5 OR #6 OR #7 OR #8 OR #9 OR #10 OR #11 OR #12 #17 #13 OR #14 #18 #15 AND #16 AND #17	2
**Embase**	#1(‘transgender’/exp OR ‘transgender’) AND[ embase ]/ lim #2 (transsexual: ab,ti OR transsexualism:ab,ti OR transgenderism:ab,ti) AND [embase]/lim #3 ‘transsexualism’/exp AND [ embase ]/ lim #4 (‘hormone substitution’/exp OR ‘hormone substitution’) AND [ embase ]/ lim #5 ‘hormone replacement ‘: ab ,ti AND [ embase ]/ lim #6 (‘estrogen therapy’/exp OR ‘estrogen therapy’) AND [ embase ]/ lim #7 ‘estrogen replacement ‘: ab ,ti AND [ embase ]/ lim #8 ‘testosterone’/exp AND [ embase ]/ lim #9 ‘testosterone therapy ‘: ab ,ti AND [ embase ]/ lim #10 ‘progesterone’/exp AND [ embase ]/ lim #11 ‘progesterone therapy ‘: ab ,ti AND [ embase ]/ lim #12 (tumor *: ab ,ti OR neoplasm:ab,ti OR neoplasia*: ab,ti OR cancer*: ab,ti ) AND [ embase ]/ lim #13 ‘neoplasm’/ mj AND [ embase ]/ lim # 14 # 1 OR #2 OR #3 # 15 # 4 OR #5 OR #6 OR #7 OR #8 OR #9 OR #10 OR #11 # 16 # 12 OR #13 # 17 # 14 AND #15 AND #16	212
**Virtual** **Health Library**	(Transgender people OR transgender persons OR transgender people OR transsexualisme OR transsexualidade OR transexualismo OR transexual) AND (hormone replacement therapy OR hormone replacement therapy OR hormone replacement therapy OR hormone replacement) AND (tumeurs OR neoplasms OR neoplasias OR câncer OR cancer OR tumor*) AND ( db :(“LILACS” OR “IBECS” OR “WPRIM” OR “BINACIS” OR “LIPECS” OR “BDENF” OR “SES-SP”))	101
**Epistemonikos**	( title :(Transgender OR Transsexual OR Transsexualism) OR abstract:(Transgender OR Transsexual OR Transsexualism)) AND (title:(“Hormone Replacement” OR Progesterone OR Testosterone OR Estrogen) OR abstract:(“Hormone Replacement” OR Progesterone OR Testosterone OR Estrogen)) AND (title:(Cancer OR tumor OR tumors ) OR abstract:(Cancer OR tumor OR tumors ))	22
**Total**		476

The following inclusion criteria were considered: studies that analyzed the risk of developing cancer in transgender people undergoing hormone therapy, published in Portuguese, English, and Spanish without date restrictions; and study designs included systematic reviews, cohort studies, non-randomized and randomized clinical trials. Cross-sectional studies were not included due to the difficulty in establishing a causal relationship between exposure and outcome. Three reviewers independently screened titles and abstracts according to predefined criteria, aided by the Rayyan reference manager. Data extraction was conducted independently by three blinded reviewers and organized in an Excel spreadsheet under topics such as author, year, study type, intervention description, cancer type reported, target population characteristics, results, and study quality assessment. A narrative synthesis was performed due to methodological heterogeneity and outcome measure variability, which prevented a meta-analysis.

The methodological quality of the included studies was assessed using the Joanna Briggs Institute’s critical appraisal tools. Checklists were used for cohort and case-control studies. Quality assessments were conducted by one reviewer and verified by a second.

## RESULTS

A total of 476 publications were identified in the electronic databases. Of these, 380 were screened by title and abstract after duplicate removal, with 359 excluded. A total of 21 publications were accessed for full-text eligibility, and 5 publications were ultimately included in this rapid review.

**Figure 1:**
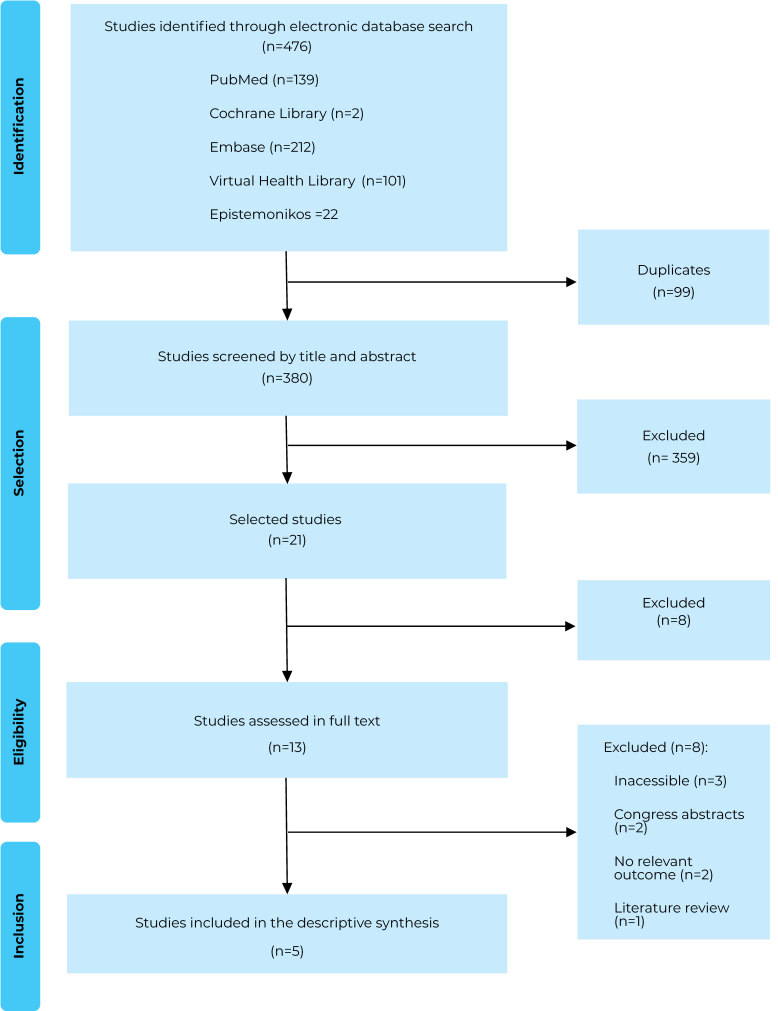
Process of including studies in the review

Figure 1 shows the flowchart of the review with the numbers of studies included and excluded at stage of the review. The full list of studies excluded after full-text reading, along with the reasons for exclusion, can be found in the review protocol (https://doi.org/10.17605/OSF.IO/F7DK8). Of the studies included for this review, 4 were cohort studies;^17- 20^ 1 was a case-control study;^21^ 2 studies were published in 2013;^17, 21^ 1 was published in 2019;^18^ 1, in 2020; ^19^ and 1, in 2021, ^20^ with sample sizes ranging from 447 to 3,489 individuals (Table 1).

**Table 1:** Characteristics of the included studies

Study	Location	Design	Date	N (% women)	Quality score
Gooren 2013^17^	Netherlands	Cohort	1975-2006	3,102 (74.3)	8/11
Block 2019^18^	Netherlands	Cohort	1972-2016	3,489 (64.7)	8/11
Williams 2020^19^	United States	Cohort	2009-2019	0^a^	8/11
Baker 2021^20^	United States	Cohort	2013-2019	447 (no information provided)	7/11
Wierckx 2013^21^	Belgium	Case-control	1986-2012	352 transgender (60.7); 1,033 cisgender (59.9)	8/10

a) Study involving transgender and cisgender men.

The outcomes analyzed in transgender people undergoing hormone therapy were diverse: 1 study analyzed the prevalence of cancer^;^ 21 2 studies analyzed the incidence of cancer;^17,^ 18 and 2 studies, the rates of epithelial cell abnormalities and histopathological change^s19,^ 20 (Table 2).

**Table 2:** Outcomes analyzed in the included studies

Study	Type of cancer	Outcomes
Gooren 2013^17^	Breas	Cancer incidence in transgender men: 4.1/100,000, (95%CI 0.8;13.0) Transgender women: 5.9/100,000 (95%CI 0.5;27.4)
Block 2019^18^	Breast	Cancer incidence in combined transgender women and men: 43/100,000
Williams 2020^19^	Not specified	Atypical squamous cells of undetermined significance: Transgender men: 5.9%
Baker 2021^20^	Breast	Histopathological alterations Atypical lesions: 11/447
Wierckx 2013^21^	Not specified	Cancer prevalence Transgender men: 0 Transgender women 28/1,000 people

Regarding cancer incidence, 2 Dutch cohort studies did not identify a significant increase in new cases of breast cancer in the transgender population.^17, 18^ The incidence rate of breast cancer in transgender women was lower than that of cisgender women, but within the expected range for cisgender men. The breast cancer incidence rate in transgender men was lower than expected for cisgender women but within the expected range for cisgender men. Breast cancer incidence in both transgender groups was comparable to that of male breast cancers. 

Another study ^18^ identified a higher risk of breast cancer in transgender women compared to cisgender men. It also identified a lower risk in transgender men compared to cisgender women. Most of the tumors identified were of ductal origin with estrogen and progesterone receptors, and 8.3% were attributed to the protein responsible for the mammary cell growth. Another important finding was that breast cancer in transgender individuals was diagnosed at a younger age compared to cisgender women. The absolute risk of breast cancer in transgender people remained low; therefore, breast cancer screening should follow the guidelines for cisgender people. ^18^

A study from Belgiu^m^ 21 showed that the cancer rate in transgender individuals was similar to or lower than that of cisgender men and women in the control group – none of the transgender men developed cancer during the follow-up period. The transgender women in this study had undergone hormone therapy for an average of 7.7 years (ranging from 3 months to 35 years); of these, 42.5% had received oral estrogen. The majority of transgender women (65%) had undergone orchiectomy. Transgender men had been on testosterone replacement therapy for an average of 9.4 years (ranging from 3 months to 49 years), and 65% of these individuals had received intramuscular testosterone treatment using a mixture of testosterone esters. Among transgender men, 86% had undergone hysterectomy and/or oophorectomy. ^21^

Two U.S. studies^19, 20^ on epithelial cells and histopathological changes also showed no increased risk of cancer in transgender people. One of them analyzed Pap smear results in a group of cisgender women and transgender men. No significant relationship was found between the duration of testosterone therapy and the presence of transitional metaplastic cells or small cells in the Pap smears of transgender patients. The difference in epithelial cell abnormality rates between the two cohorts was not statistically significant. ^19^

A total of 447 surgical breast specimens from gender-confirming chest contouring surgeries were reviewed and histopathological findings were compared between transgender men and male-centered gender nonconforming individuals who did and did not receive testosterone therapy. Intramuscular testosterone was administered to 85.6% of the sample, followed by 7.9% who used transdermal testosterone gel or cream. The majority of individuals who had undergone hormone therapy for at least 12 months had moderate lobular atrophy (p-value<0.001). Atypical lesions were detected in 2.5% of individuals who had undergone hormone therapy. The study’s results indicated that hormone therapy did not lead to clinically significant changes in breast morphology (i.e., an increased frequency of atypical lesions or carcinoma), even after more than 12 months of use. ^20^

Although the study population was small and young, with findings based on a limited sample, routine breast cancer screening of residual tissue following chest contouring surgery for transgender men and male-centered gender nonconforming individuals was considered important. ^20^ In addition to cancer, other morbidities such as venous thrombosis and/or pulmonary embolism, acute myocardial infarction, and cerebrovascular disease were tracked. Five percent of transgender women had venous thrombosis and/or pulmonary embolism. Transgender women had more myocardial infarctions than cisgender women in the control group (p-value 0.001), but the proportion was similar to that of men in the control group. The prevalence of cerebrovascular disease was higher in transgender women than in cisgender men in the control group (p-value 0.03). Rates of myocardial infarction and cardiovascular disease in transgender men were similar to those in cisgender individuals in the control group. The prevalence of type 2 diabetes was higher in both transgender men and women when compared to their respective controls.^21^

## DISCUSSION

This review included five studies and aimed to identify scientific evidence on the risk of cancer in transgender people undergoing hormone therapy. Two of these studies^17, 19^ indicated an overall increased risk of neoplasia in this group, while the others^16,18, 20^ did not find this association. The studies included in this rapid systematic review presented robust methodologies with considerable population samples and well-defined inclusion and exclusion criteria. Breast cancer was the neoplasm most likely to develop in transgender individuals using hormone therapy.^17, 19^ It is noteworthy that the magnitude of this risk was not thoroughly evaluated in this review. Further research is needed to confirm and quantify this association.

Reliable epidemiological data remains scarce despite advances in studies in this field. Transgender people can develop cancer in organs related to the sex assigned at birth, as well as in newly formed organs.^17, 19^

The increased cancer risk in transgender people, compared with the cisgender population, is multifactorial. This includes the high prevalence of sexually transmitted infections, increased exposure to risk factors such as smoking and alcohol consumption, and lack of adequate access to cancer screening.^11^ Several publications have identified cancer as a priority in studies on transgender people health and have recognized the importance of large-scale population-based studies.^11, 21^

Estrogen and testosterone therapy in transgender people must be continuously monitored due to the relative risk of other morbidities, such as the development of cardiovascular diseases.^20^ Doses and methods of administration may also vary during hormone replacement, which reinforces the need for close monitoring by healthcare teams for transgender people on hormone therapy.^22^

Transgender people may present a distinct risk of developing chronic conditions and health conditions, as well as risk factors for these diseases and health conditions.^22^Specific recommendations for transgender people regarding cancer and other comorbidities are necessary for better comprehensive health care. However, there are uncertainties about cancer screening protocols for transgender people.^22^

The transsexualization process within the Brazilian National Health System is constantly marked by the lack of network coordination. Consequently, transgender people are often responsible for navigating their own therapeutic pathway, which frequently leads to seeking care in alternative spaces. These factors result in the abusive use of hormones, a lack of assistance from health professionals and treatment abandonment. 

This situation worsens the health conditions of transgender people and contributes to the normalization of care neglect and the perpetuation of inequities. 23

One of the limitations of this rapid review was the unavailability of two systematic reviews due to lack of access to full texts and the exclusion of cross-sectional studies. Rapid reviews are conducted using some shortcuts to shorten the execution time and, therefore, no contact was made with the authors to request the full text and analyze bias. The exclusion of cross-sectional studies is justified by the difficulty in establishing causal relationships in this type of design.

The absence of a meta-analysis prevents a more accurate and robust analysis of data, which limits the ability to generate accurate estimates of the effects of hormone therapy on neoplasms. Although the studies included in this review were not set in the Brazilian context, the findings of this review may support the development of public policies regarding the follow-up of transgender people undergoing hormone therapy.

Few studies were included, and neither a systematic review nor a randomized clinical trial, which are considered the highest levels of scientific evidence, were included. The evidence retrieved showed that certain types of studies, such as randomized clinical trials, are still lacking to better answer the research question.

The main objective of this review was to investigate the risk of developing cancer in transgender individuals undergoing hormone therapy. Although the results of the included studies were heterogeneous, making it difficult to establish a definitive conclusion on the increased risk, the need for further research in this area is evident, especially in the Brazilian context. Given the specificities of the Brazilian transgender population and the existing knowledge gaps, future studies should deepen the investigation of risk factors, the most prevalent types of cancer, and the best practices for monitoring the health of this population. A potential association with an increased incidence of cancer should not be used at this time as a contraindication for hormone therapy, but should support the development of recommendations for the follow-up of the transgender population undergoing hormone therapy. This aims at population screening and early diagnosis of cancer.
