# Supplementary material for: Hormone therapy and cancer risks in transgender people: a systematic review
Source: Epidemiol Serv Saude. 2025 Jan 10;33(spe1):e2024319. doi: 10.1590/S2237-96222024v33e2024319.especial.en (PMC11734654; doi:10.1590/S2237-96222024v33e2024319.especial.en)
Supplement: Translation

# Translation: A hormonioterapia e os riscos de câncer em pessoas transgênero: revisão sistemática

**DOI:** 10.1590/S2237-96222024v33e2024319.especial.pt

**Authors:** Liza Yurie Teruya Uchimura, Tatiana Yonekura, Mabel Fernandes Figueiró, Jeane Roza Quintans, Patrícia Freire, Fernando Henrique de Albuquerque Maia

**Affiliations:** 1 Hospital do Coração, São Paulo, SP, Brasil; 2 Fundação de Ensino e Pesquisa em Ciências da Saúde, Brasília, DF, Brasil; 3 Ministério da Saúde, Secretaria de Atenção Especializada à Saúde, Brasília, DF, Brasil; 4 Universidade de São Paulo, Faculdade de Medicina, Departamento de Medicina Preventiva, SP, Brasil.

**Keywords:** Pessoas Transgênero, Neoplasias Malignas, Hormonal, Hormonioterapia, Revisão Sistemática

## Abstract

**Objetivo:**

Identificar as evidências disponíveis sobre o risco de desenvolver câncer em pessoas transgênero que se tratam por meio de hormonioterapia.

**Métodos:**

Trata-se de uma revisão sistemática rápida nas bases de dados PubMed, Embase, Biblioteca Virtual em Saúde, Cochrane Library e Epistemonikos. A triagem e a extração de dados foram conduzidas por revisores independentes na plataforma Rayyan. A extração dos dados foi realizada por 3 revisores independentes. Utilizamos os *checklists* do Instituto Joanna Briggs específicos para estudos de coorte e caso-controle para avaliar a qualidade metodológica dos estudos incluídos.

**Resultados:**

Foram incluídos 5 estudos, sendo 4 estudos de coorte e 1 caso-controle. O risco de pessoas transgênero desenvolverem câncer em hormonioterapias foi identificado por 2 estudos e não identificado por 3 estudos.

**Conclusão:**

Apesar de estudos com grandes amostras e critérios de seleção rigorosos, a literatura não apresenta consenso sobre a associação entre a hormonioterapia e o desenvolvimento de câncer em indivíduos transgênero.

## Contribuições do estudo

**Principais resultados:**

Identificaram-se 5 estudos publicados entre 2013 e 2021. O risco de pessoas transgênero desenvolverem câncer em hormonioterapias foi identificado por 2 estudos, porém não há um consenso claro para recomendações.

**Implicações para os serviços:**

Contribui com a atenção integral à saúde das pessoas transgênero. Isso favorece a melhor decisão no processo de hormonioterapia por essas pessoas e amplia a possibilidade de escolha dos recursos disponibilizados na rede de atenção à saúde.

**Perspectivas:**

A revisão destaca a necessidade de mais estudos randomizados e de longo prazo para avaliar os riscos e benefícios da hormonioterapia em pessoas transgênero, em diferentes subgrupos, como idade, tempo de utilização e tipo de hormônios.

## INTRODUÇÃO

O conceito de transgênero ou não conformista de gênero caracteriza pessoas cuja identidade de gênero difere daquela atribuída ao nascimento.^1,2^ Essa divergência pode levar à transição, que inclui tratamentos hormonais e cirurgias de afirmação de gênero, com o objetivo de alinhar o corpo à identidade de gênero. Aproximadamente, 0,6% da população dos Estados Unidos se identifica como transgênero.^3^ No Brasil, aproximadamente, 2% da população é transgênero.^4^

A Associação Mundial Profissional para a Saúde Transgênero enfatiza a necessidade de cuidados clínicos individualizados aos transexuais, aos transgêneros e àqueles que não se identificam pelo gênero de nascimento. Em linha com as diretrizes dessa associação, que defende a importância dos cuidados individualizados, estudos têm demonstrado que a cirurgia de redesignação sexual apresenta melhores resultados em serviços com equipes multidisciplinares especializadas em cuidados com pessoas transgênero.^5^

A Política Nacional de Saúde Integral de Lésbicas, *Gays*, Bissexuais, Travestis e Transexuai^s6^ e as normativas do processo transexualizador no Sistema Único de Saúd^e7^ identificam como fundamental a garantia de acesso ao acompanhamento ambulatorial para o atendimento à pessoa transgênero no processo transexualizador. Isso evidencia a importância do trabalho multiprofissional no Sistema Único de Saúde.^6-8^

O número de serviços e de profissionais da saúde capazes de propor um cuidado em saúde integral para as pessoas transgênero ainda é insuficiente, o que demonstra um vazio assistencial no território nacional para essa população. Até 2022, somente 11 serviços no Brasil eram habilitados para atendimento na atenção especializada no processo transexualizador. Em 2023 foram habilitados mais 10 serviços.^9^

A terapia hormonal aprimora as características sexuais secundárias para que fiquem congruentes com a identidade de gênero do indivíduo. Pessoas transgênero podem receber hormônios esteroides, prescritos por profissionais de saúde, para reduzir o sofrimento psicológico e induzir mudanças físicas desejadas, como redução da oleosidade da pele, gordura corporal e crescimento/perda de pelos.^10^ A hormonioterapia para efeitos de afirmação de gênero pode ser administrada em doses elevadas e durante um período de décadas.^3^

O risco de câncer decorrente da terapia hormonal em pessoas transgênero é uma área de considerável preocupação.^11^ Esse potencial risco muitas vezes é utilizado como justificativa para contraindicar ou suspender a hormonioterapia. O uso de hormônios, especialmente o estrogênio, pode influenciar o desenvolvimento de células cancerígenas, o que aumenta o risco de certos tipos de câncer, como o de mama. É importante ressaltar que o risco varia de acordo com diversos fatores, incluindo o tipo de hormônio, a dose e a duração do tratamento. Outros fatores de risco, como histórico familiar e hábitos de vida, também podem contribuir para o desenvolvimento da doença. 

Os benefícios da hormonioterapia para a saúde mental e o bem-estar das pessoas transgênero são inegáveis. É essencial identificar o risco, a fim de impulsionar, direcionar e informar as políticas de saúde pública e aumentar a sensibilização para as questões que afetam as pessoas transgênero. 

Esta revisão sistemática rápida tem como objetivo identificar as evidências disponíveis sobre o risco de desenvolver câncer no uso de hormonioterapia por pessoas transgênero.

## MÉTODOS

Tratou-se de uma revisão rápida, seguindo as etapas de acordo com o Cochrane Rapid Reviews Methods Grou^p12^ para investigar o risco de desenvolvimento de câncer em pessoas transgênero que se tratam por meio de hormonioterapia. A metodologia da revisão rápida visa maior agilidade na condução da revisão, buscando gerar o menor impacto possível na qualidade e no viés do estudo.^13^ A pergunta de pesquisa desta revisão foi construída com base no acrônimo PECOS (população, exposição, comparador, desfechos e tipo de estudo) (Quadro 1). Esta revisão rápida foi guiada pela pergunta: Qual o risco de pessoas transgênero desenvolverem câncer utilizando hormonioterapias?

**Quadro 1:** Pergunta de pesquisa estruturada em população, exposição, comparador, desfechos e tipo de estudo (PECOS)

P	Pessoas transgênero
E	Hormonioterapia (progesterona, testosterona, estrógeno)
C	Pessoas sem hormonioterapia
O	Risco de desenvolver qualquer tipo de câncer
S	Revisão sistemática, estudo de coorte, ensaios clínicos não randomizados, ensaios clínicos randomizados e caso-controle

Os atalhos metodológicos adotados para esta revisão foram: idioma de publicação em português, inglês e espanhol; e avaliação da qualidade metodológica realizada por um revisor com checagem por um segundo revisor. O protocolo foi publicado antes do início da seleção dos estudos no repositório *online* Zenodo.org.

A busca na literatura foi realizada em outubro de 2023, sem limitação de data, nas bases de dados eletrônicas: PubMed, Embase, Cochrane Library, Biblioteca Virtual em Saúde e Epistemonikos. As estratégias de busca foram construídas a partir do vocabulário controlado Medical Subject Headings (MeSH) do PubMed, Emtree do Embase e Descritores em Ciências da Saúde (DeCS) da Biblioteca Virtual em Saúde (BVS). Os vocabulários não controlados, como palavras-chave e sinônimos, também foram utilizados na elaboração das estratégias de busca (Quadro 2). 

**Quadro 2:** Estratégia de busca e resultado obtido em cada base bibiolográfica

Base de dados	Estratégia de busca	Resultados
**PubMed**	(((“Transgender Persons”[Mesh] OR Person, Transgender OR Transgender Person OR Transgenders OR Transgender OR Transgendered Persons OR Person, Transgendered OR Persons, Transgendered OR Transgendered Person OR Two-Spirit Persons OR Person, Two-Spirit OR Two Spirit Persons OR Two-Spirit Person OR Transsexual Persons OR Person, Transsexual OR Transsexual Person OR Transexuals OR Transexual) OR (“Transsexualism”[Mesh] OR Transgenderism)) AND ((((“Hormone Replacement Therapy”[Mesh] OR Therapy, Hormone Replacement OR Hormone Replacement Therapies OR Replacement Therapies, Hormone OR Therapies, Hormone Replacement OR Replacement Therapy, Hormone) OR (“Testosterone”[Mesh] OR “Testosterone therapy”)) OR (“Progesterone”[Mesh] OR “Progesterone therapy”)) OR (“Estrogen Replacement Therapy”[Mesh] OR Estrogen Replacement Therapies OR Replacement Therapies, Estrogen OR Therapies, Estrogen Replacement OR Therapy, Estrogen Replacement OR Replacement Therapy, Estrogen OR Estrogen Replacement OR Estrogen Replacements OR Replacements, Estrogen OR Replacement, Estrogen OR Estrogen Progestin Replacement Therapy OR Estrogen Progestin Combination Therapy))) AND (“Neoplasms”[Mesh] OR Tumor OR Neoplasm OR Tumors OR Neoplasia OR Neoplasias OR Cancer OR Cancers OR Malignant Neoplasm OR Malignancy OR Malignancies OR Malignant Neoplasms OR Neoplasm, Malignant OR Neoplasms, Malignant OR Benign Neoplasms OR Benign Neoplasm OR Neoplasms, Benign OR Neoplasm, Benign)	139
**Cochrane Library**	1 MeSH descriptor: [Transgender Persons] explode all trees #2 (Transgender Person OR Transgenders OR Transgender OR Transgendered Persons OR Person, Transgendered OR Persons, Transgendered OR Transgendered Person OR Two-Spirit Persons OR Person, Two-Spirit OR Two Spirit Persons OR Two-Spirit Person OR Transsexual Persons OR Person, Transsexual OR Transsexual Person OR Transexuals OR Transexual):ti,ab,kw #3 MeSH descriptor: [Transsexualism] explode all trees #4 (Transsexualism OR Transgenderism):ti,ab,kw #5 MeSH descriptor: [Hormone Replacement Therapy] explode all trees #6 (Therapy, Hormone Replacement OR Hormone Replacement Therapies OR Replacement Therapies, Hormone OR Therapies, Hormone Replacement OR Replacement Therapy, Hormone):ti,ab,kw #7 MeSH descriptor: [Testosterone] explode all trees #8 (“Testosterone therapy”):ti,ab,kw #9 MeSH descriptor: [Progesterone] explode all trees #10 (“Progesterone therapy”):ti,ab,kw #11 MeSH descriptor: [Estrogen Replacement Therapy] explode all trees #12 (Estrogen Replacement Therapies OR Replacement Therapies, Estrogen OR Therapies, Estrogen Replacement OR Therapy, Estrogen Replacement OR Replacement Therapy, Estrogen OR Estrogen Replacement OR Estrogen Replacements OR Replacements, Estrogen OR Replacement, Estrogen OR Estrogen Progestin Replacement Therapy OR Estrogen Progestin Combination Therapy):ti,ab,kw #13 MeSH descriptor: [Neoplasms] explode all trees #14 (Tumor OR Neoplasm OR Tumors OR Neoplasia OR Neoplasias OR Cancer OR Cancers OR Malignant Neoplasm OR Malignancy OR Malignancies OR Malignant Neoplasms OR Neoplasm, Malignant OR Neoplasms, Malignant OR Benign Neoplasms OR Benign Neoplasm OR Neoplasms, Benign OR Neoplasm, Benign):ti,ab,kw #15 #1 OR #2 OR #3 OR #4 #16 #5 OR #6 OR #7 OR #8 OR #9 OR #10 OR #11 OR #12 #17 #13 OR #14 #18 #15 AND #16 AND #17	2
**Embase**	#1(‘transgender’/exp OR ‘transgender’) AND[embase]/lim #2 (transsexual:ab,ti OR transsexualism:ab,ti OR transgenderism:ab,ti) AND [embase]/lim #3 ‘transsexualism’/exp AND [embase]/lim #4 (‘hormone substitution’/exp OR ‘hormone substitution’) AND [embase]/lim #5 ‘hormone replacement’:ab,ti AND [embase]/lim #6 (‘estrogen therapy’/exp OR ‘estrogen therapy’) AND [embase]/lim #7 ‘estrogen replacement’:ab,ti AND [embase]/lim #8 ‘testosterone’/exp AND [embase]/lim #9 ‘testosterone therapy’:ab,ti AND [embase]/lim #10 ‘progesterone’/exp AND [embase]/lim #11 ‘progesterone therapy’:ab,ti AND [embase]/lim #12 (tumor*:ab,ti OR neoplasm:ab,ti OR neoplasia*:ab,ti OR cancer*:ab,ti) AND [embase]/lim #13 ‘neoplasm’/mj AND [embase]/lim #14 #1 OR #2 OR #3 #15 #4 OR #5 OR #6 OR #7 OR #8 OR #9 OR #10 OR #11 #16 #12 OR #13 #17 #14 AND #15 AND #16	212
**Biblioteca Virtual em Saúde**	(Pessoas transgênero OR transgender persons OR personas transgénero OR transsexualisme OR transexualidade OR transexualismo OR transexual) AND (terapia de reposição hormonal OR hormone replacement therapy OR terapia de reemplazo de hormonas OR reposição hormonal OR reposição de hormônios) AND (tumeurs OR neoplasms OR neoplasias OR câncer OR cancer OR tumor*) AND ( db:(“LILACS” OR “IBECS” OR “WPRIM” OR “BINACIS” OR “LIPECS” OR “BDENF” OR “SES-SP”))	101
**Epistemonikos**	(title:(Transgender OR Transsexual OR Transsexualism) OR abstract:(Transgender OR Transsexual OR Transsexualism)) AND (title:(“Hormone Replacement” OR Progesterone OR Testosterone OR Estrogen) OR abstract:(“Hormone Replacement” OR Progesterone OR Testosterone OR Estrogen)) AND (title:(Cancer OR tumor OR tumours) OR abstract:(Cancer OR tumor OR tumours))	22
**Total**		476

Foram considerados critérios de inclusão: estudos que analisaram o risco de desenvolvimento de câncer em pessoas transgênero que se tratam por meio de hormonioterapia, publicados em qualquer ano e nos idiomas português, inglês e espanhol; e estudos com desenhos de revisão sistemática, estudo de coorte, ensaios clínicos não randomizados e ensaios clínicos randomizados. Os estudos transversais não foram incluídos pela dificuldade de estabelecer uma relação causal entre exposição e desfecho. A triagem dos títulos e resumos das referências identificadas foi realizada de forma independente por três revisores, seguindo os critérios de elegibilidade preestabelecidos, com auxílio do gerenciador de referências Rayyan. Realizou-se a extração dos dados por três revisores de forma cega e independente. Os dados foram organizados em uma planilha de Excel, seguindo a categorização definida com os tópicos: autor, ano, tipo de estudos incluídos na revisão, descrição da intervenção, tipo de câncer relatado, características da amostra (público-alvo), resultados e avaliação da qualidade metodológica dos estudos. Realizou-se a síntese dos dados de forma narrativa, considerando a presença de diversidades metodológica e de medidas de desfechos dos estudos incluídos. Isso impossibilitou o desenvolvimento de meta-análise com o cálculo de estimativas de efeito.^14^

Para a avaliação da qualidade metodológica dos estudos incluídos, foram utilizadas as ferramentas de avaliação crítica do Instituto Joanna Briggs.^15^ Utilizou-se a lista de verificação para estudos de coorte,^16^ e para o estudo de caso-controle, a lista de verificação para estudos de caso-controle.^15^ A avaliação foi realizada por um revisor e confirmada por um segundo revisor. 

## RESULTADOS

Foram identificadas 476 publicações nas bases de dados eletrônicas. Destas, após a remoção das duplicadas, 380 foram triadas por leitura de título e resumo, tendo sido excluídas 359, e acessadas, para a elegibilidade da leitura de texto completo, 21 publicações. Ao todo foram incluídas 5 publicações nesta revisão rápida. 

Na Figura 1, é possível observar o fluxograma da revisão com os números de estudos incluídos e excluídos por etapa da revisão. No protocolo dessa revisão, (https://doi.org/10.17605/OSF.IO/F7DK8) está a lista completa de estudos excluídos após leitura na íntegra com o motivo de exclusão. Dos estudos incluídos para esta revisão, 4 apresentaram desenho de coorte;^17-20^ 1 era caso-controle;^21^ 2 estudos foram publicados em 2013;^17,21^ 1 foi publicado em 2019;^18^ 1, em 2020;^19^ e 1, em 2021,^20^ amostra entre 447 e 3.489 indivíduos (Tabela 1). 

**Figura 1:** Processo de inclusão dos estudos na revisão

**Tabela 1:** Características dos estudos incluídos

**Estudo**	**Local**	**Delineamento**	**Data**	**n (% mulheres)**	**Escore de qualidade**
Gooren 2013^17^	Holanda	Coorte	1975-2006	3.102 (74,3)	8/11
Block 2019^18^	Holanda	Coorte	1972-2016	3.489 (64,7)	8/11
Williams 2020^19^	Estados Unidos	Coorte	2009-2019	0^a^	8/11
Baker 2021^20^	Estados Unidos	Coorte	2013-2019	447 (não informado)	7/11
Wierckx 2013^21^	Bélgica	Caso-controle	1986-2012	352 transgênero (60,7); 1.033 cisgênero (59,9)	8/10

a) estudo com homens transgênero e cisgênero.

Os desfechos analisados nas pessoas transgênero em uso de hormonioterapia foram diversos: 1 estudo analisou a prevalência de câncer;^21^ 2 estudos analisaram a incidência de câncer;^17,18^ e 2 estudos, as taxas de anormalidade de células epiteliais e alterações histopatológicas^19,20^ (Tabela 2).

**Tabela 2:** Desfechos analisados nos estudos incluídos

**Estudo**	**Tipo de câncer**	**Desfechos**
Gooren 2013^17^	Mama	Incidência de câncer em homens transgênero: 4,1/100.000, (IC_95_% 0,8;13,0) Mulheres transgênero: 5,9/100.000, (IC_95_% 0,5;27,4)
Block 2019^18^	Mama	Incidência de câncer em mulheres e homens transgênero combinados: 43/100.000
Williams 2020^19^	Não especificado	Células escamosas atípicas de significado indeterminado: Homens transgênero: 5,9%
Baker 2021^20^	Mama	Alterações histopatológicas Lesões atípicas: 11/447
Wierckx 2013^21^	Não especificado	Prevalência de câncer Homens transgênero: 0 Mulheres transgênero 28/1.000 pessoas

Em relação ao desfecho de incidência, 2 coortes holandesas não identificaram aumento significativo de novos casos de câncer de mama na população transgênero.^17,18^ A taxa de incidência de câncer de mama em mulheres transgênero foi abaixo da que foi comparada com as mulheres cisgênero, porém dentro das expectativas para homens cisgênero. A taxa de incidência de câncer de mama para homens transgênero foi inferior ao esperado para mulheres cisgênero, mas dentro do esperado para o câncer de mama em homens cisgênero. A incidência do câncer de mama em ambos os grupos transgênero é comparável a dos cânceres de mama masculinos.^17^

Outro estud^o18^ identificou maior risco de câncer de mama em mulheres transgênero em comparação com homens cisgênero. Identificou também menor risco em homens transgênero em comparação com mulheres cisgênero. A maioria dos tumores identificados era de origem ductal com receptores de estrogênio e progesterona, e 8,3% eram devido à proteína responsável pelo crescimento das células mamárias. Outro importante resultado está no momento do diagnóstico de câncer de mama em indivíduos transgênero com idade mais jovem, quando comparado com mulheres cisgênero. O risco absoluto de câncer de mama em pessoas transgênero permaneceu baixo; portanto, o rastreamento do câncer de mama deve seguir as diretrizes para pessoas cisgênero.^18^

Estudo da Bélgica^21^ mostrou que a taxa de câncer em pessoas transgênero foi semelhante ou inferior à de homens e mulheres cisgênero do grupo controle – nenhum dos homens transgênero desenvolveu câncer durante o período de acompanhamento. As mulheres transgênero desse estudo realizaram hormonioterapia por uma média de 7,7 anos (variação de 3 meses a 35 anos); destas, 42,5% receberam estrogênio oral. A maioria das mulheres transgênero (65%) foi submetida à orquiectomia. Homens transgênero estiveram em terapia de reposição de testosterona por uma média de 9,4 anos (variação de 3 meses a 49 anos), e 65% desses indivíduos realizaram tratamento intramuscular de testosterona com uma mistura de ésteres de testosterona. Dessa amostra de homens transgênero, 86% foram submetidos à histerectomia/ovariectomia.^21^

Dois estudos norte-americanos^19,20^ sobre células epiteliais e alterações histopatológicas não mostraram também maior risco de câncer em pessoas transgênero. Um deles analisou resultados de exames de Papanicolau em um grupo de mulheres cisgênero e homens transgênero. Não foi encontrada nenhuma relação significativa entre a duração da terapia com testosterona e a presença das células transicionais de metaplasia e pequenas em exames de Papanicolau dos pacientes transgênero. A diferença entre as taxas de anormalidade das células epiteliais nas duas coortes não foi estatisticamente significativa.^19^

Foram revisadas 447 amostras cirúrgicas de mama provenientes de cirurgias de contorno torácico de confirmação de gênero e comparadas com os achados histopatológicos entre homens transgênero e em indivíduos não conformes de gênero centrados no masculino que receberam e não receberam terapia de testosterona. Os 85,6% da amostra realizaram administração de testosterona intramuscular, seguidos de 7,9% que usaram gel ou creme transdérmico de testosterona. A maioria dos indivíduos que receberam hormonioterapia por um período mínimo de 12 meses apresentou atrofia lobular moderada (p-valor<0,001). As lesões atípicas foram detectadas em 2,5% dos pacientes que receberam hormonioterapia. Os resultados do estudo indicaram que a hormonioterapia não resultou em alterações clinicamente significativas na morfologia mamária (isto é, aumento da frequência de lesões atípicas ou carcinoma), mesmo quando é realizado por mais de 12 meses.^20^

Apesar de a população do estudo ser pequena e jovem, além de achados em uma amostra reduzida, considerou-se importante o rastreamento rotineiro de câncer de mama em tecido residual após cirurgia de contorno torácico para homens transgênero e em indivíduos não conformes de gênero centrados no masculino.^20^ Rastrearam-se, além do câncer, outras morbidades como trombose venosa e/ou embolia pulmonar, infarto agudo do miocárdio e doenças cerebrovasculares. Cinco por cento das mulheres transgênero apresentaram trombose venosa e/ou embolia pulmonar. Mulheres transgênero tiveram mais infartos do miocárdio do que as mulheres cisgênero do grupo controle (p-valor=0,001), mas uma proporção semelhante em comparação com os homens do grupo controle. A prevalência de doença cerebrovascular foi maior em mulheres transgênero do que em homens cisgênero do grupo controle (p-valor=0,03). As taxas de infarto do miocárdio e doenças cardiovasculares em homens transgênero foram semelhantes às dos indivíduos cisgênero do grupo controle. A prevalência de diabetes tipo 2 foi maior tanto em homens como em mulheres transgênero quando comparada com seus respectivos controles.^21^

## DISCUSSÃO

Esta revisão incluiu cinco estudos e teve o intuito de identificar evidências científicas sobre o risco de câncer em pessoas transgênero submetidas à hormonioterapia. Dois desses estudo^s17,19^ apontaram um aumento no risco geral de neoplasias neste grupo, enquanto os demais^16,18,20^ não encontram essa associação. Os estudos incluídos nesta revisão sistemática rápida apresentaram métodos robustos com amostra populacional considerável e com critérios de inclusão e exclusão bem-definidos. O câncer de mama é a neoplasia que apresentou maior propensão de desenvolvimento em pessoas transgênero em uso de hormonioterapia.^17,19^ Destaca-se que a magnitude desse risco não foi avaliada em profundidade nesta revisão. São necessárias pesquisas adicionais para confirmar e quantificar essa associação. 

Dados epidemiológicos confiáveis ainda são escassos apesar dos avanços dos estudos na área. Pessoas transgênero podem desenvolver câncer em órgãos relacionados ao sexo atribuído no nascimento, bem como em órgãos recém-formados.^17,19^

O risco aumentado de câncer em pessoas transgênero, em comparação com a população cisgênero, é multifatorial. Isso inclui a maior prevalência de infecções sexualmente transmissíveis, o aumento da exposição a fatores de risco, como o tabagismo e o consumo de álcool, e a falta de acesso adequado ao rastreio de neoplasias.^11^ Várias publicações identificaram o câncer como prioridade na investigação sobre a saúde de pessoas transgênero e reconheceram a importância de estudos em grande escala populacional.^11,21^

O tratamento com estrógenos e testosterona das pessoas transgênero deve seguir em constante monitoramento devido ao risco relativo de outras morbidades, como o desenvolvimento de doenças cardiovasculares.^20^ Doses e formas de administração também podem apresentar modificações no decorrer da reposição hormonal, o que reforça o acompanhamento próximo da equipe de saúde aos indivíduos transgênero em uso de hormonioterapia.^22^

As pessoas transgênero podem apresentar risco diferenciado de ocorrência de condições crônicas e agravos não transmissíveis, bem como dos fatores de risco para essas doenças e agravos.^22^ Recomendações específicas para as pessoas transgênero em relação ao câncer e a outras morbidades são necessárias para um melhor cuidado da saúde de forma integral. Entretanto, existem incertezas sobre os protocolos de rastreamento para câncer em pessoas transgênero.^22^

O processo transexualizador no Sistema Único de Saúde constantemente é marcado pela ausência da articulação da rede. Isso resulta em pessoas transgênero responsáveis por traçar o próprio percurso terapêutico, o que leva muitas vezes à busca por assistência em espaços alternativos. Esses fatores resultam no uso abusivo de hormônios, na desassistência dos profissionais de saúde e no abandono do tratamento. A descrição desse cenário piora as condições de saúde das pessoas transgênero e favorece a naturalização de negligências assistenciais e produtoras de iniquidades.^23^

Uma das limitações desta revisão rápida foi a indisponibilidade de duas revisões sistemáticas devido à falta de acesso ao texto completo e à exclusão de estudos transversais. As revisões rápidas são realizadas com alguns atalhos para abreviar o tempo de execução e, assim, não foram feitas consultas aos autores para solicitação do texto completo e análise de viés. A exclusão de estudos transversais se justifica pela dificuldade em estabelecer relações causais nesse tipo de delineamento. 

A ausência de uma metanálise impede uma análise mais precisa e robusta dos dados, o que limita a capacidade de gerar estimativas precisas dos efeitos da hormonioterapia sobre as neoplasias. Apesar de os estudos incluídos nesta revisão não estarem no contexto brasileiro, os achados desta revisão poderão subsidiar a construção de políticas públicas acerca do acompanhamento das pessoas transgênero em uso de hormonioterapia. 

Houve a inclusão de poucos estudos e não foram incluídos a revisão sistemática e o ensaio clínico randomizado, que são delineamentos de estudos considerados como as melhores evidências científicas. As evidências recuperadas mostraram que ainda faltam determinados tipos de estudos, como os ensaios clínicos randomizados, para melhor responder à pergunta de pesquisa. 

Esta revisão teve como objetivo principal investigar o risco de desenvolvimento de câncer em pessoas transgênero em uso de hormonioterapia. Embora os resultados dos estudos incluídos tenham sido heterogêneos, não sendo possível estabelecer uma conclusão definitiva sobre o aumento do risco, a necessidade de mais pesquisas nessa área é evidente, em especial no contexto brasileiro. Considerando-se as particularidades da população transgênero brasileira e as lacunas de conhecimento existentes, estudos futuros devem aprofundar a investigação sobre os fatores de risco, os tipos de câncer mais prevalentes e as melhores práticas para o acompanhamento da saúde dessa população. Uma possível associação com aumento de incidência de câncer não deve ser utilizada neste momento como contraindicação para a hormonioterapia, mas deve subsidiar a construção das recomendações para acompanhamento da população transgênero em uso de hormonioterapia. Isso visa ao rastreamento populacional e ao diagnóstico precoce do câncer.
